# Direct Oral Anticoagulants and Bleeding Management Following Tooth Extractions—A Prospective Cohort Study

**DOI:** 10.3390/dj12090279

**Published:** 2024-08-30

**Authors:** Rossana Izzetti, Chiara Cinquini, Marco Nisi, Marco Mattiozzi, Monica Marotta, Antonio Barone

**Affiliations:** Department of Surgical, Medical and Molecular Pathology and Critical Care Medicine, University Hospital of Pisa, 56126 Pisa, Italy

**Keywords:** direct-acting oral anticoagulants, tooth extractions, dentistry, operative, oral surgical procedure, post-operative hemorrhage, oral hemorrhage

## Abstract

The aim of the present study was to assess the occurrence of intra-, peri-, and post-operative bleeding following tooth extractions in patients treated with direct oral anticoagulants (DOACs). Consecutive patients requiring at least one dental extraction were enrolled. The DOAC regimen was either maintained or suspended. Patients were classified in subgroups depending on the number of teeth extracted per procedure (≤3 or >3), the need for flap elevation, and the performance of osteotomy. Bleeding was recorded intra-operatively; peri-operatively at 20, 40, 60, and 80 min after the procedure; and daily in the first seven days following tooth extractions. Forty-nine patients treated with DOACs (17 with rivaroxaban, 16 with apixaban, 8 with edoxaban, and 8 with dabigatran) were enrolled. Of them, 33 refrained from DOAC administration pre-operatively. The performances of >3 teeth extractions, flap elevation, and osteotomy were significantly associated with higher bleeding rates (*p* < 0.05). In patients treated with rivaroxaban and apixaban, bleeding episodes were more frequent. Although DOAC treatment may increase the rates of intra-operative, peri-operative, and post-operative bleeding, the recorded episodes were mild and manageable. DOAC suspension may reduce peri-operative bleeding, while no effect could be observed for post-operative bleeding.

## 1. Introduction

Direct oral anticoagulants (DOACs) have gained popularity in recent years due to their predictable pharmacokinetic profile and perceived convenience compared to traditional anticoagulants, like warfarin, eliminating the need for routine dose titration [1]. These medications exert their anticoagulant effects by targeting specific factors involved in the coagulation cascade, thereby preventing thrombus formation. Specifically, dabigatran directly inhibits thrombin formation, while apixaban, rivaroxaban, and edoxaban are factor Xa inhibitors [2].

The inherent anticoagulant activity of DOACs poses a risk of bleeding complications, particularly in the peri-operative period, following dental surgical procedures, such as tooth extractions [3].

At present, different protocols can be found in the literature regarding the management of patients treated with DOACs in the dental setting. While guidelines have been developed to address the challenges faced by patients on vitamin K antagonists (VKAs) during dentoalveolar procedures [4], limited evidence is available on DOACs. Perry et al. [5] standardized the approach for patients under VKAs by checking INR within 72 h prior to the procedure, without the need for specific intervention for values < 4, and by adding local hemostatic measures and 5% tranexamic acid mouthwash administration four-times daily for 48 h after the procedure. Douketis et al. [6] examined the bleeding outcomes of 460 individuals treated with oral anticoagulants who required dental procedures. No significant differences were noted in outcomes between the warfarin and dabigatran treatment groups, although the performance of urgent procedures increased the risk of bleeding or thromboembolism. Clinical practice guidelines by Valenzuela-Mencia et al. [7] suggested that patients on DOACs may face a higher risk of post-operative bleeding after dental extractions compared to healthy individuals, although the paucity of the data available limited the strength of the recommendations. However, it should be highlighted that single-tooth extraction poses a limited risk of post-operative bleeding, thus providing an indication for the non-discontinuation of DOACs for simple, single, dental extractions [8]. Moreover, it is estimated that only a minority of patients who undergo dentoalveolar surgery will re-attend for repeated applications of local measures for hemostasis, in order to manage persistent bleeding, thus highlighting that modifications to DOAC administration are seldom required [9].

The management of post-operative bleeding in patients on DOAC therapy requires a delicate balance between minimizing the risk of thromboembolic events and controlling hemorrhages. Unlike vitamin K antagonists, such as warfarin, DOACs have a rapid onset of action and a shorter half-life, making the reversal of their anticoagulant effects more challenging [10]. Traditional reversal agents, such as vitamin K or fresh frozen plasma, are not effective against DOACs. Instead, specific antidotes have been developed for some DOACs, such as idarucizumab for dabigatran and andexanet alfa for rivaroxaban and apixaban, which can rapidly neutralize their anticoagulant effects in emergency situations [11].

Nevertheless, the decision to administer reversal agents must be carefully weighed against the patient’s individual risk of thrombosis and the severity of bleeding [11]. In cases of minor bleeding following a tooth extraction, local measures such as pressure hemostasis, topical hemostatic agent application, and tranexamic acid mouthwashes may be sufficient to achieve hemostasis without the need for reversal agents [12]. However, in the presence of significant or persistent bleeding, especially in patients at a high risk of thromboembolic events, the use of specific antidotes may be warranted to expedite hemostasis and minimize morbidity and mortality [11]. Furthermore, the timing of tooth extractions in relation to DOAC administration is an important consideration when preventing post-operative bleeding. Given the short half-life of DOACs, a transient discontinuation of therapy may be sufficient to reduce the risk of bleeding without significantly increasing the risk of thrombosis [12].

Assessing post-operative bleeding risk in patients on DOACs undergoing dental extractions is crucial due to the increasing use of these medications, which present a complex challenge in balancing bleeding and thromboembolic risks during surgery [13]. Current evidence is overall limited, with varied management protocols. The aim of the present study was therefore to assess the bleeding risk associated to tooth extractions in patients treated with direct oral anticoagulants.

## 2. Materials and Methods

### 2.1. Study Protocol

**Figure 1:**
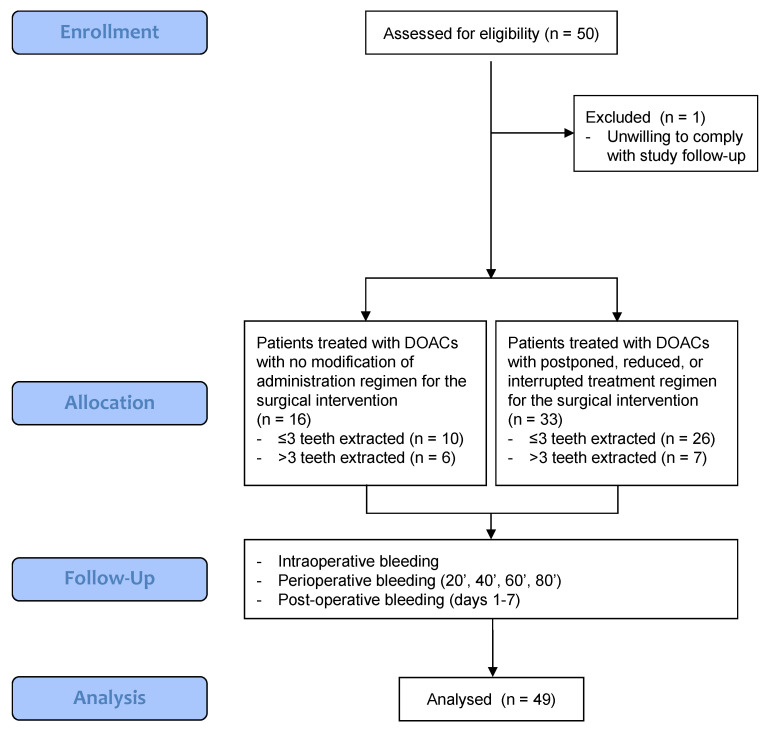
CONSORT 2010 flow diagram.

The study was single-center, non-randomized, clinical trial with a 1-week follow-up duration. The protocol was approved by the institutional review board of the University Hospital of Pisa (Ethics committee North-West Tuscany area, approval no. 17796/2020). The study was conducted according to the principles outlined in the Declaration of Helsinki on experimentation involving human subjects and was recorded on a public registry of clinical trials (www.clinicaltrials.gov, registration code NCT06365242). This article adheres to the STROBE (Strengthening the reporting of observational studies in epidemiology) guidelines [7]. The flow diagram of the study is shown in Figure 1.

### 2.2. Patients Enrolment

Consecutive patients referred to the Unit of Dentistry and Oral Surgery for dental extraction between 2021 and 2023 were enrolled in this study. All the study participants signed an informed consent form to be included in the study. Patient data underwent a rigorous anonymization process, ensuring that all personal identifiers were removed or encrypted to protect confidentiality and comply with the data protection regulations.

Inclusion criteria were: (i) age > 18 years; (ii) patients under pharmacological treatment with DOACs; (iii) patients requiring at least one tooth extraction; and (iv) patients willing to take part in the study and to sign informed consent forms.

Patients below 18 years of age, patients treated with antiplatelet therapy or vitamin K antagonists, and patients who suffered from congenital or acquired coagulopathies (e.g., hemophilia, coagulation factors deficiency, von Willebrand disease, thrombocytopenia, or cirrhosis) were excluded.

All the patients included were prescribed the following blood tests: complete blood count, prothrombin time (PT), partial thromboplastin time (PTT), and INR.

### 2.3. Study Groups

At baseline, patients were assigned to the following groups according to the administration regimen decided by their cardiologist:

- Group 1: Patients treated with DOACs with no modification of administration regimen for the surgical intervention.
- Group 2: Patients treated with DOACs with postponed, reduced, or interrupted treatment regimens for the surgical intervention. Indication of DOACs suspension was provided by the cardiologist or the general practitioner based on the clinical conditions of the patient.

Patients were stratified depending on the medication received (rivaroxaban, apixaban, edoxaban, and dabigatran). Data on systemic health, DOACs assumption dose, body mass index (BMI), and smoking habit were collected for each patient. Vital parameters, including blood pressure, heart rate, saturation, respiratory rate, body temperature, and glycemia, were registered on the day of intervention in all patients. All data were registered in a dedicated case report form (CRF).

### 2.4. Surgical Intervention

**Figure 2:**
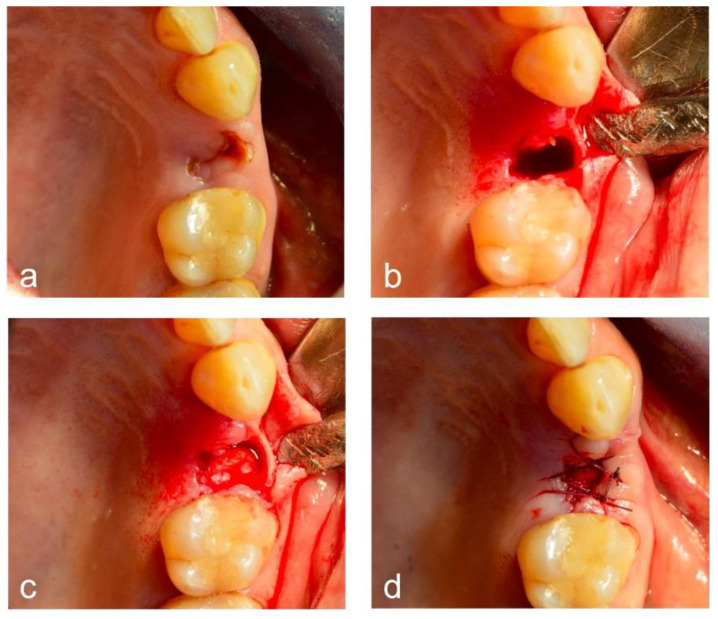
Tooth 2.5 requiring extraction (**a**), flap elevation and tooth removal (**b**), collagen sponge application (**c**), and suture placement (**d**).

Tooth extractions were conducted under standardized conditions in all patients, using a minimally invasive surgical technique (Figure 2).

Briefly, local anesthesia with mepivacaine 2% + epinephrine 1:100.000 was performed. Tooth extractions were conducted with the support of elevators and forceps. A full-thickness flap was elevated in the case of a difficult extraction. Patients in both groups 1 and 2 were further divided in two groups, depending on the number of teeth extracted, with a cut-off value of 3 teeth (≤3 teeth and >3 teeth). The choice of this cut-off was based on previous literature reporting that the extraction of up to three teeth can be considered a low-risk intervention [14]. The post-extractive socket was managed with careful local debridement and saline rinsing, and a collagen sponge was placed inside the alveolus. Soft tissues were then repositioned with single, interrupted, absorbable sutures. No additional hemostatic measures were performed at this stage. Following surgery, antibiotic treatment (amoxicillin at 2 gr/die or clindamycin at 1200 mg/die in the case of an allergy to penicillin) was prescribed for 6 days if indicated, depending on systemic health conditions. Co-dispensation of antibiotics to patients on DOACs was not considered a factor increasing the risk of bleeding [15,16], conversely to what is reported for warfarin [17]. Acetaminophen at 1 gr was prescribed for the management of pain symptoms.

### 2.5. Bleeding Episodes’ Registration

The presence of intra-operative bleeding was defined as the development of active bleeding hindering the visibility of the surgical area.

Peri-operative bleeding was registered as oozing or active bleeding that could not be controlled with local hemostatic measures and/or compression with a gauze occurring within the first 80 min following surgery, which was registered every 20 min. If bleeding was detected, a gauze with tranexamic acid was applied. After 20 min of gauze compression, oxidized cellulose and additional sutures were applied if active bleeding was still present. In cases of prolonged bleeding, diathermocoagulation was performed.

Post-operative bleeding was defined as the bleeding or oozing of the surgical wound beyond 12 h post-operatively, leading to the formation of a hematoma of the oral soft tissue and requiring intervention or even hospitalization.

### 2.6. Follow-Up

Following tooth extractions, the patients received instructions for the management of the surgical wound and a questionnaire to be filled during the post-operative week. The questionnaire investigated the number of bleeding episodes, how they were managed, and if additional medications were needed for pain management. At 7 days follow-up, patients were re-evaluated to rule out the presence of complications (such as oedema, pain, suppuration, and alveolar osteitis), and the suture was removed. The evaluation of post-operative bleeding was performed by an operator not involved in the surgical treatment.

### 2.7. Sample Size Estimation

The aim was to evaluate the incidence of bleeding episodes following tooth extractions in patients treated with different DOACs. Based on the study by Berton et al. [8] reporting bleeding episodes needing additional management in 20% of patients treated with DOACs, a sample size of at least 28 subjects (significance level a = 0.05, power = 0.9, and difference in proportion = 0) was required to obtain valid and reliable results. It was decided to enroll 50 patients to manage potential dropouts.

### 2.8. Statistical Analysis

The statistical analysis was performed by a dentist expert in biostatistics who was not involved in the surgical procedures nor in the data acquisition.

Descriptive statistics were reported as means and standard deviations, and the Shapiro–Wilk test was employed to verify the normal distribution of data, and parametric or non-parametric tests were used accordingly. In case of quantitative or ordinal variables, a comparison between groups was performed using the Kruskal–Wallis test, while for multiple comparisons, the Mann–Whitney test and Wilcoxon signed rank test were employed. Dichotomic variables were analyzed using chi-squared test or Fisher’s exact tests. All the statistical analyses were performed using XLStat software (XLSTAT 2023.1.4 (1408) statistical and data analysis solution, Addinsoft, Paris, France). All CRFs must be completed at least at 95% to be included in the analysis, and missing data were managed by using regression imputation.

## 3. Results

### 3.1. Patient Population

Forty-nine patients (21 females, 42.9%) receiving 136 tooth extractions completed the study. One patient was excluded due to a lack of compliance to the study follow-up. Mean age was 72.2 years (SD 8.3 years). Seventeen patients were treated with rivaroxaban (20 mg/die n = 16, 15 mg/die n = 1), 16 patients with apixaban (10 mg/die n = 9, 5 mg/die n = 7), 8 patients received edoxaban (30 mg/die n = 2, 60 mg/die n = 6), and 8 patients received dabigatran (300 mg/die n = 5, 220 mg/die n = 3). On average, the patients were treated with DOACs for 38 months (SD 27 months). Demographic data are shown in Table 1.

Thirty-three patients (67% of the sample) interrupted the treatment with DOACs prior to surgery, with a mean suspension time of 28.48 ± 13.04 h at the time of surgery. Treatment suspension was prescribed by the cardiologist to 14 patients, by the general practitioner to 4 patients, in 11 cases by other specialists, and 4 patients autonomously decided not to take the medication. All patients refrained from DOAC therapy for at least 24 h following surgery.

The diseases requiring treatment with DOACs were atrial fibrillation (55.1%), deep vein thrombosis (24.5%), ischemic cardiomyopathy (18.3%), pulmonary embolism (16.3%), valvular dysfunction (12.2%), and heart failure (8%). Hypertension was present in 40.8% of patients, while 24.5% of patients suffered from type II diabetes. Mean BMI was 28.3 (±5.5) kg/m^2^, and therefore overweight. Six patients were current smokers (12.2%) and 24 patients (49%) were former smokers. Mean value of systolic blood pressure was 133 mmHg (SD 23.8 mmHg), mean diastolic blood pressure was 79 mmHg (SD 11.5 mmHg), heart rate was 77.6 bpm (SD 14.4 bpm), saturation was 96% (SD 1.7%), respiratory rate was 15 (SD 2), body temperature was 36.4 °C (SD 0.4 °C), and glycemia was 121.4 mg/dl (49 mg/dl). At baseline, statistically significant differences in vital parameters were registered for body temperature and glycemia.

Blood testing (Table 2) highlighted a significant difference in the PT ratio for rivaroxaban compared to apixaban (*p* = 0.038) and edoxaban (*p* = 0.03). Patients treated with rivaroxaban also had a significantly higher PTT compared to patients treated with other DOACs (*p* = 0.001). Mean eGFR was 82.61 mL/min, indicating mild renal impairment.

### 3.2. Features of Tooth Extraction Procedures

In 36 patients (73.5%), ≤3 teeth were extracted. In patients receiving less than three dental extractions per procedure, statistically significant lower rates of peri-operative bleeding were encountered. Differences in post-operative bleeding depending on the number of extracted teeth were registered only at T2 and T7. Removing adjacent teeth did not influence bleeding rates.

In 26.5% of procedures, a surgical flap was raised, and in 30.6%, osteotomy was performed, accounting for 57.1% of complex extractions. Peri-operative bleeding was higher in the case of flap elevation, although a statistically significant difference was registered only at 20 min from the intervention. Post-operative bleeding rates were significantly higher at all timepoints for clinical cases who required a flap elevation.

### 3.3. Intra-Operative Bleeding

Intra-operative bleeding was registered in 27 patients (55.1%). Twelve patients were treated with apixaban, nine with rivaroxaban, three with edoxaban, and three with dabigatran. Patients who received more than three tooth extractions bled in 69.2% of cases, while in patients with less than three teeth extracted, bleeding occurred in 50% (*p* < 0.001). No statistical difference was registered when comparing patients who suspended the use of DOACs and patients who did not.

### 3.4. Peri-Operative Bleeding

Peri-operative bleeding at 20 min post-operatively occurred in 28 patients (57.1%), specifically in 14 patients receiving apixaban, 6 patients receiving edoxaban, 5 patients treated with rivaroxaban, and 3 patients receiving dabigatran. Peri-operative bleeding at 20 min statistically differed in patients treated with apixaban compared to rivaroxaban (Table 3). Bleeding in patients on DOACs was more frequent in the extraction of posterior teeth compared to anterior teeth (81% vs. 64%) and in complex extractions compared to simple extractions (26% vs. 13%).

At 40 min, bleeding was still present in 19 patients (38.8% out of 49 patients, 7 apixaban, 6 rivaroxaban, 4 edoxaban, and 2 dabigatran). Fourteen patients experienced bleeding at 60 min (28.6% out of 49 patients, 5 rivaroxaban, 4 apixaban, 3 edoxaban, and 2 dabigatran), and 8 patients at 80 min (16.3% out of 49 patients, 5 rivaroxaban, and 3 apixaban).

No statistical differences in terms of peri-operative bleeding risk were registered at any timepoint when comparing patients who suspended DOAC administration and patients not varying the treatment regimen.

### 3.5. Post-Operative Bleeding

Post-operative bleeding episodes peaked at day 1 post-op, for 23 patients (46.9%; 11 rivaroxaban, 7 apixaban, 4 edoxaban, and 1 dabigatran). At day 2, 11 bleeding episodes were registered (22.4%; 7 rivaroxaban, 3 apixaban, and 1 edoxaban). At day 3, four bleeding episodes occurred (8.2%, four rivaroxaban, four apixaban, and four edoxaban). From days 4 to 7, bleeding was present only in patients treated with rivaroxaban, with two episodes at days 4 and 5 and one episode at days 6 and 7, in the absence of statistically significant differences.

Stratification of the results per treatment group highlighted a statistically significant difference at T1 and T2 between patients treated with rivaroxaban and dabigatran (*p* = 0.016 at T1, *p* = 0.023 at T2).

Although, in the absence of statistical significance, a trend for post-operative bleeding was noted in patients who did not discontinue DOAC therapy at T1 and T2, while delayed bleeding (T3-T7) was observed in patients refraining from DOACs assumption (Table 4).

## 4. Discussion

According to the present results, around 50% of patients treated with DOACs experienced intra- and early peri-operative bleeding following tooth extractions, while post-operative bleeding peaked at day 1 post-operatively. The decision to continue or temporarily stop DOACs did not appear to significantly impact the risk of bleeding following dental extractions, as similar bleeding rates were noted regardless of whether DOAC therapy was interrupted or maintained, provided that appropriate local hemostatic measures were employed. This suggests that the inherent properties of DOACs, such as their predictable pharmacokinetics and short half-lives, allow for safe dental procedures without the need for discontinuation.

In patients undergoing more than three tooth extractions in the same session, intra-operative, peri-operative, and post-operative bleeding were higher with statistically significant differences at all time points (except at T1), regardless of the DOAC therapeutic protocol employed. While a cut-off of three teeth extracted was also recognized by Lupi et al. [14], Labadibi et al. [18] reported that DOAC suspension could be avoided in cases requiring fewer than four tooth extractions at one time with the use of adjunctive local hemostatic measures. Cocero et al. [19] suggested scheduling the timing of extractions at least 4 h after the last DOAC intake. The authors also recommended to avoid the extraction of two or three contiguous premolars and molars in one session. However, the extraction of contiguous teeth did not represent a risk factor in our sample.

Indeed, the occurrence of bleeding episodes in patients treated with DOACs is influenced by many factors, including the type of medication received. The higher rates of bleeding episodes encountered in patients treated with rivaroxaban may be ascribed to the fact that rivaroxaban slightly prolongs prothrombin time and partial thromboplastin time [20,21], while apixaban is a highly selective factor Xa (FXa) inhibitor of both free and bound FXa, as well as prothrombinase, independent of antithrombin III for the prevention and treatment of thromboembolic diseases [22]. Overall, in our sample, bleeding events occurred in around 50% of patients, while in the study by Yagyuu et al. [23], incidences of post-extraction bleeding (10.4%) among extractions that involved DOACs occurred in 10% of patients. Hiroshi et al. [24] reported an even lower incidence of bleeding episodes, with frequencies of post-extractive hemorrhage of 1.65% for dabigatran and 3.41% for rivaroxaban.

Patel et al. [25] encountered post-operative bleeding in 13% of procedures, and in half of the reported cases, the bleeding was self-limiting and did not require additional interventions. The authors concluded post-operative bleeding requiring additional local hemostatic measures can be anticipated in a 6% of cases, and DOAC regime modification should be evaluated case per case [25].

There have been conflicting results in the literature regarding the need for DOAC administration modification during surgical dental procedures. Initially, discontinuing DOACs 24 h prior to surgery or maintaining treatment regimens were considered the two feasible options, however, more recently, it has been hypothesized that delaying this could represent a valuable option in cases of invasive dental procedures [26].

Despite the thromboembolic risk associated with DOAC interruption being low, DOAC interruption should be performed only in cases of a high expected risk of bleeding. Conversely, in cases of a low bleeding risk, a short-term interruption is deemed unnecessary and does not require heparin bridging, which is associated with increased bleeding in the absence of a decrease in the risk of thromboembolism [27].

The protocol by van Diermen et al. [28] suggests the performance of tooth extractions at least 1–3 h following DOAC administration, while the European Heart Rhythm Association [29] recommends reaching trough concentration (12 h after DOAC administration) prior to performing an extraction. Brennan and colleagues [30] conducted a prospective cohort study in the absence of changes in DOACs’ administration and evaluated pharmacological variables related to timing, ranging from the last dose and anticoagulant serum concentration. Bleeding rates among different types of DOACs were comparable (apixaban, 39%; rivaroxaban, 37%; dabigatran, 27%). Pre-extraction blood levels of apixaban and dabigatran were similar between subjects who experienced bleeding and those who did not, while pre-extraction blood levels of rivaroxaban were approximately two-times higher in those who experienced bleeding. The majority of patients on rivaroxaban therapy who experienced bleeding had taken rivaroxaban during the morning of the extraction, 82% (95% CI, 52.3–94.9), compared to 18% (95% CI, 5.1–47.7) who had instead taken the last dose of rivaroxaban the night before dental avulsion.

Overall, it appears that tooth extractions could be performed safely on patients treated with DOACs by applying local hemostatic measures, without interrupting or modifying the therapeutic regimen, as DOACs do not significantly increase the risk of post-extraction bleeding [8,23,31,32]. However, it should be borne in mind that an extreme variability in DOAC discontinuation can be observed, even in the same cohort of patients, as reported by Miller and Miller [33]. In fact, the authors reported a variability in temporary DOAC interruptions ranging between 12 and 120 h prior to the oral surgery procedure. The continuation or discontinuation of DOACs in patients undergoing oral surgical procedures remains controversial and requires further studies to extrapolate the results [2].

In our sample, treatment with rivaroxaban and apixaban was associated with a higher bleeding risk. DOAC therapy suspension before the procedure did not influence post-operative bleeding rates. Tooth extractions involving flap raising were associated with increased bleeding risk at 20 min post-operation and from T1 to T7. Intra-operative and peri-operative bleeding rates were also higher in cases requiring an osteotomy at 20 and 40 min from the end of the procedure.

Although it is not possible to draw conclusive associations, given the small sample size, bleeding in patients on DOACs occurred more frequently in extractions of posterior dental elements compared to anterior ones and in complex extractions compared to simple extractions.

The present study has some limitations. Firstly, the sample size was limited, while a larger number of patients and better balancing between groups would have provided more definitive evidence to establish a protocol for action in such patients. In the study, bleeding risk was assessed in the overall population of patients treated with DOACs, either suspended or not, and no further stratification depending on the type of medication was performed, as a discrepant number of patients per medication was encountered. Patient inclusion may have led to selection bias due to the presence of different protocols for DOAC treatment management, as most patients refrained from DOAC administration in the pre-operative period, although not following the same suspension protocol. Moreover, the timing of intervention with respect to the last DOAC administration was not taken into account due to the fact that the patients received their medications at different times of the day; therefore, it would have been difficult to schedule and stratify patients depending on DOACs’ half-lives in such a limited sample. The lack of DOAC quantifications represented a limitation as well. The intensity of anticoagulation at the time of surgery and post-operatively was not assessed through DOAC activity assays, such as specific calibrated Anti-Factor-Xa- assays or simple Anti-Xa-assays. Further studies evaluating this aspect are required in the literature. The variability in the surgical procedures, including number of teeth extracted, flap raising, and performance of osteotomy, may hinder accurate comparisons among patients and the generalizability of the results. Although the sample was heterogeneous and limited in dimensions, thus hindering the drawing of firm conclusions, the results of this study still appear relevant, as the close monitoring of the post-operative period allowed us to evaluate the dynamics of different DOACs following dental extractions. Indeed, further research is needed to provide clear, evidence-based guidelines to optimize patient outcomes and ensure safe management during dental procedures. This would not only improve patient outcomes, but also provide peace of mind to both patients and healthcare providers when navigating the complexities of anticoagulation therapy in the context of dental procedures.

## 5. Conclusions

Patients treated with new oral anticoagulants show comparable rates of intra-operative, peri-operative, and post-operative bleeding. Bleeding recorded both in the peri-operative and post-operative periods was mild and manageable with gauze compression. In our sample, the suspension of anticoagulant therapy did not influence peri-operative bleeding or post-operative bleeding. However, larger studies with better balancing between variables are required to corroborate our findings.

## Figures and Tables

**Table 1:** Demographic data.

Variables	Total Sample (n = 49)	Rivaroxaban (n = 17)	Apixaban (n = 16)	Edoxaban (n = 8)	Dabigatran (n = 8)	*p* Value
Age (years; mean [SD])	72.2 (8.3)	72 (5.6)	72.8 (9.7)	70.8 (11)	73.1 (8.3)	0.807
Gender (females [%])	21 (42.9)	9 (53.0)	6 (37.5)	4 (50.0)	2 (25.0)	0.560
BMI (kg/m^2^; mean [SD])	28.3 (5.5)	29.2 (4.2)	28 (5.3)	26.4 (2.1)	32.7 (8.6)	0.133
Non-smokers [%])	43 (87.8)	12 (70.6)	8 (50.0)	5 (62.5)	2 (25.0)	0.133
Systolic pressure (mean [S]D)	133 (23.8)	134.4 (21.3)	125.7 (26.3)	136.5 (12.5)	140 (31.6)	0.299
Diastolic pressure (mean [SD])	79 (11.5)	82.6 (12.5)	76.6 (11.7)	75.3 (8.0)	79.3 (11.4)	0.293
Body temperature (mean [SD])	36.4 (0.4)	36.1 (0.4)	36.4 (0.3)	36.7 (0.3)	36.3 (0.3)	0.026
Glycemia (mean [SD])	121.4 (49.0)	119.3 (30.0)	98 (11.4)	119.9 (28.7)	173.9 (93.9)	0.004
Heart rate (mean [SD])	77.6 (14.4)	75.8 (10.1)	72.3 (11.1)	83 (24.5)	74.4 (15.0)	0.712

**Table 2:** Blood testing values and comparison between groups.

Variables (Mean [SD])	Total Sample (n = 49)	Rivaroxaban (n = 17)	Apixaban (n = 16)	Edoxaban (n = 8)	Dabigatran (n = 8)	*p* Value
Red blood cells (10^6^/mm^3^)	4.33 (0.38)	4.31 (0.37)	4.51 (0.66)	4.06 (0.69)	4.43 (0.60)	0.313
Hemoglobin (g/dL)	12.55 (2.07)	12.29 (2.09)	13.35 (1.99)	12.13 (1.64)	13.28 (1.73)	0.431
Platelets (10^3^/mm^3^)	216.47 (58.46)	226.00 (53.53)	233.0 (64.99)	217.50 (110.71)	236.13 (55.48)	0.728
PT ratio	1.34 (0.17)	1.37 (0.15)	1.25 (0.22)	1.18 (0.12)	1.20 (0.18)	0.014
INR	1.31 (0.15)	1.31 (0.15)	1.25 (0.23)	1.25 (0.03)	1.21 (0.09)	0.318
aPTT (sec)	36.57 (3.75)	37.06 (3.89)	32.06 (3.52)	32.14 (3.41)	46.95 (8.75)	<0.001
aPTT ratio	1.04 (0.14)	1.06 (0.13)	1.02 (0.08)	1.22 (0.01)	1.51 (0.31)	<0.001
Fibrinogen (mg/dL)	367.07 (81.52)	366.33 (55.80)	351.5 (86.41)	351.20 (63.83)	403.29 (101.92)	0.299
Creatinine (mg/dL)	0.98 (0.36)	0.98 (0.35)	0.92 (0.27)	0.87 (0.26)	1.07 (0.29)	0.062
Estimated glomerular filtration rate	82.61 (10.63)	77.23 (13.48)	84.46 (13.43)	84.59 (16,92)	90.31 (21.17)	0.646

**Table 3:** Peri-operative bleeding and comparison between groups.

Bleeding	Total Sample (n = 49)	Rivaroxaban (n = 17)	Apixaban (n = 16)	Edoxaban (n = 8)	Dabigatran (n = 8)	*p* Value
20′ min (n [%])	28 (57.1)	5 (29.4)	14 (87.5)	6 (75.0)	3 (37.5)	0.010
40′ min (n [%])	19 (38.8)	6 (35.3)	7 (43.75)	4 (50.0)	2 (25.0)	0.731
60′ min (n [%])	14 (28.6)	5 (29.4)	4 (25.0)	3 (37.5)	2 (25.0)	0.928
80′ min (n [%])	8 (16.3)	5 (29.4)	3 (18.75)	0 (0.0)	0 (0.0)	0.157

**Table 4:** Post-operative bleeding rates and comparison between suspension or continuation of OAC therapy.

Bleeding	DOAC Continued (n = 16)	DOAC Suspended (n = 33)	*p* Value
T1 (n [%])	8 (50.0)	15 (45.6)	0.357
T2 (n [%])	5 (31.3)	6 (18.2)	0.304
T3 (n [%])	1 (6.3)	3 (9.1)	0.733
T4 (n [%])	1 (6.3)	1 (3.0)	0.587
T5 (n [%])	0 (0.0)	2 (6.1)	0.315
T6 (n [%])	0 (0.0)	1 (3.0)	0.482
T7 (n [%])	0 (0.0)	2 (6.1)	0.315

## Data Availability

Due to ethical reasons, supporting data is not available.
